# Necroptosis in CNS diseases: Focus on astrocytes

**DOI:** 10.3389/fnagi.2022.1016053

**Published:** 2023-01-27

**Authors:** Elena V. Mitroshina, Mariia Saviuk, Maria V. Vedunova

**Affiliations:** Institute of Biology and Biomedicine, National Research Lobachevsky State University of Nizhni Novgorod, Nizhny Novgorod, Russia

**Keywords:** necroptosis, RIP1 kinase, RIP3 kinase, astrocyte, neurodegenerative disease, ischemia

## Abstract

In the last few years, necroptosis, a recently described type of cell death, has been reported to play an important role in the development of various brain pathologies. Necroptosis is a cell death mechanism that has morphological characteristics similar to necrosis but is mediated by fundamentally different molecular pathways. Necroptosis is initiated by signaling through the interaction of RIP1/RIP3/MLKL proteins (receptor-interacting protein kinase 1/receptor-interacting protein kinase 3/mixed lineage kinase domain-like protein). RIPK1 kinase is usually inactive under physiological conditions. It is activated by stimulation of death receptors (TNFR1, TNFR2, TLR3, and 4, Fas-ligand) by external signals. Phosphorylation of RIPK1 results in the formation of its complex with death receptors. Further, complexes with the second member of the RIP3 and MLKL cascade appear, and the necroptosome is formed. There is enough evidence that necroptosis plays an important role in the pathogenesis of brain ischemia and neurodegenerative diseases. In recent years, a point of view that both neurons and glial cells can play a key role in the development of the central nervous system (CNS) pathologies finds more and more confirmation. Astrocytes play complex roles during neurodegeneration and ischemic brain damage initiating both impair and protective processes. However, the cellular and molecular mechanisms that induce pathogenic activity of astrocytes remain veiled. In this review, we consider these processes in terms of the initiation of necroptosis. On the other hand, it is important to remember that like other types of programmed cell death, necroptosis plays an important role for the organism, as it induces a strong immune response and is involved in the control of cancerogenesis. In this review, we provide an overview of the complex role of necroptosis as an important pathogenetic component of neuronal and astrocyte death in neurodegenerative diseases, epileptogenesis, and ischemic brain damage.

## Introduction

For many years, apoptosis was considered to be the only form of regulated cell death, while necrosis was regarded as a process of unregulated accidental cell death (ACD). The initial characterization of apoptosis and necrosis focused on morphological features. Apoptotic cells shrivel and demonstrate nuclear condensation and membrane vesicles, while necrotic cells swell and rupture. Further research showed that apoptosis depends on the activity of one or more members of the cysteine protease family (caspases). This cell death is caused by the ligation of specific cell surface receptors, DNA damage, excessive reactive oxygen species (ROS), and multiple types of cellular stress.

After identifying the major molecular pathways of apoptosis, it was also found that apoptosis-inducing stimuli cause caspase-independent cell death with morphological features similar to necrosis, including cell swelling, organelle dysfunction, and plasma membrane rupture (Chen et al., 2019a). The discovery of new genetic and biochemical pathways of regulation, as well as specific chemical inhibitors of necrosis, determined that some processes of necrosis can also be considered as a molecularly controlled regulated form of cell death. Regulated necrosis includes several cell death modalities: necroptosis, parthanatos, ferroptosis or oxytosis, mitochondrial permeability transition (MPT) dependent necrosis, pyroptosis, and pyronecrosis, netosis. Necroptosis mediated by receptor-interacting protein kinase 3 (RIPK3) and its substrate, the pseudokinase mixed lineage kinase domain-like protein (MLKL), is the most characterized form of regulated necrosis (Sun and Wang, 2014). Unlike apoptosis, necroptosis is not associated with caspases (Holler et al., 2000; Datta et al., 2020).

## Mechanism of necroptosis

The molecular mechanism of necroptosis has been described relatively recently (Sun and Wang, 2014). Receptor-interacting serine/threonine-protein kinase 1 (RIPK1) was found to play a key role in the induction and course of necroptosis. Activation of specialized cell receptors triggers necroptosis. These receptors include the tumor necrosis factor receptor (TNFR1) by TNF (Laster et al., 1988), death receptors (e.g., Fas/FasL) (Holler et al., 2000), Toll-like receptors (Kaiser et al., 2013) and cytosolic nucleic acid sensors such as RIG-I (circulating in cytosol) and STING (on the membrane of the endoplasmic reticulum), which induce the release of type-I interferon (IFN-1) and TNF and thus promoting necroptosis (Brault et al., 2018). The cGAS-STING and RIG-I-MAVS molecular cascades are critical cytosolic receptor systems that recognize and respond to DNA and RNA nucleic acids, respectively, protecting the cell from invading microbial pathogens (Zevini et al., 2017). Most of these pathways trigger nuclear factor kappa B (NfκB)-dependent pro-inflammatory and survival signals. However, additional inhibition of the proteolytic enzyme caspase-8 triggers the necroptosis pathway.

**FIGURE 1:**
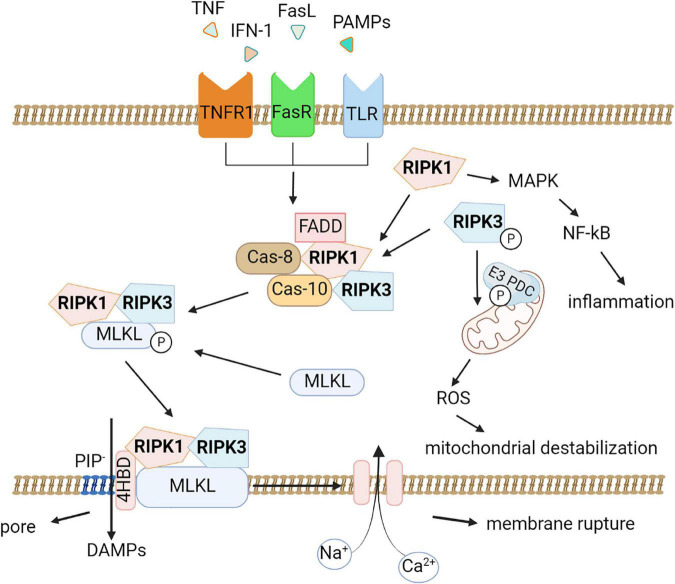
Molecular pathways of necroptosis. Activation of the tumor necrosis factor receptor (TNFR1), death receptors, Toll-like receptors triggers necroptosis. An oligomeric complex including RIPK1, Fas-associated death domain protein (FADD), caspase-8, and caspase-10 is formed and if caspase-8 activity is absent, RIPK1 recruits and phosphorylates RIPK3 to form a ripoptosome complex. Further the RIPK1/RIPK3 complex recruits and phosphorylates MLKL, thereby forming necrosomes. MLKL forms pores in the plasma membrane through an interaction between a positively charged site in the 4-helical bundle domain (4HBD) and negatively charged phosphatidylinositol phosphates (PIP) present on the membrane then the cell dies. DAMPs (HMGB1, IL-1α, ATP) can be released during this process. Also, necroptosis possibly leads to destabilization of mitochondria and the production of reactive oxygen species (ROS). RIPK1 promotes the expression of pro-inflammatory genes by activating the mitogen-activated protein kinase (MAPK) signaling pathway and the transcription factor NF-κB.

RIPK1 is recruited as part of an oligomeric complex including Fas-associated death domain protein (FADD), caspase-8, and caspase-10 (Tenev et al., 2011). In the absence of caspase-8 activity, RIPK1 recruits and phosphorylates RIPK3 to form a complex termed the ripoptosome (Li et al., 2012). RIPK1/RIPK3 complex recruits and phosphorylates MLKL, thereby forming necrosomes (Figure 1; Murphy et al., 2013; Liu et al., 2019b). Early studies suggested that the RIPK1/RIPK3/MLKL complex causes mitochondrial destabilization and subsequent cell death (Wang et al., 2012); however, this hypothesis was refuted (Murphy et al., 2013). To date, two pathways of cell death under the action of MLKL are considered possible. First, MLKL may be a plasma membrane platform for opening calcium or sodium ion channels, thereby allowing ion influx, cell swelling, and rupture (Chen et al., 2014). Other studies suggest that MLKL itself forms pores in the plasma membrane through an interaction between a positively charged site in the 4-helical bundle domain (4HBD) and negatively charged phosphatidylinositol phosphates (PIP) present on the membrane (Quarato et al., 2016).

Moreover, MLKL seems to be affected by other factors. For example, it was demonstrated that phosphorylation of MLKL by RIPK3 alone is not sufficient for its action on the membrane. In addition, MLKL requires the interaction of its N-terminal domain with highly phosphorylated inositol phosphate (IP) (Dovey et al., 2018).

Although mitochondrial damage and ROS production are not considered necroptosis activators, a recent study by Yang and colleagues showed that RIPK3 instead has a downstream effect on mitochondria. RIPK3 directly phosphorylates and activates the E3 subunit of pyruvate dehydrogenase complex and promotes aerobic respiration and production of mitochondrial ROS (Yang et al., 2018b). This finding may explain the connection between necroptosis and mitochondrial destabilization.

It is known that the externalization of phosphatidylserine (PS) to the outer surface of the plasma membrane during apoptosis is necessary for the recognition and absorption of dying cells (the “eat me” signal) and for the modulation of the immune response. It is of great interest whether there is a similar pathway in the development of necroptosis. In a number of cell lines, it has been shown that PS is externalized into the outer plasma membrane of necroptotic cells before the integrity of the plasma membrane is lost. In four different models of phagocytosis, necroptotic cells with externalized PS were shown to be phagocytosed more efficiently than viable cells but less efficiently than apoptotic cells. In addition, another feature of cell death that has been suggested to distinguish apoptosis from inflammatory-lytic forms of cell death is the formation of “apoptotic bodies”. These extracellular vesicles (EV) are 0.5 to 2 μm in size and contain nucleic acids, proteins, and organelles that can serve as damage-associated molecular patterns (DAMPs) when released into the environment. Extracellular vesicle secretion is an important mechanism used by cells to release biomolecules. It has recently been shown that necroptotic cells also secrete smaller (∼0.2–0.8 μm) EVs before cell viability or membrane integrity loss occurs. These EVs, called “necroptotic bodies,” exhibited PS on the membrane’s outer surface (like apoptotic cells). Some of them also contain proteins, including pMLKL and RIPK3. Unlike “apoptotic bodies,” “necroptotic bodies” do not appear to contain DNA. These necroptotic bodies can probably modulate the inflammatory response of surrounding cells (Edry-Botzer and Gerlic, 2017; Gupta et al., 2022). It remains to be investigated whether “necroptotic bodies” can undergo phagocytosis.

Another important aspect of the development of necroptosis is the active generation of damage-associated molecular patterns (DAMPs) during necroptotic cell death (Degterev et al., 2005). Once oligomerized MLKL binds to the cell membrane, causing its permeabilization, there is a massive release of various intracellular components, including DAMPs such as high-mobility group box (HMGB)1 and histones as well as heat shock proteins, members of the IL-1 family and ATP, which further initiate and exacerbate the development of inflammatory processes, the so-called sterile inflammation (Murai et al., 2018). This explains the role of necroptosis in the development of neuroinflammation in various neurodegenerative diseases such as Alzheimer’s disease, Parkinson’s disease, multiple sclerosis, and amyotrophic lateral sclerosis (Yuan et al., 2019). Several studies suggested that RIPK1 may also directly control inflammatory gene expression and induce inflammation independent of necroptosis-induced DAMP release. For example, RIPK1 was confirmed to promote the expression of pro-inflammatory genes by activating the mitogen-activated protein kinase (MAPK) signaling pathway and the transcription factor NF-κB (Li et al., 2022a).

Thus, by virtue of the immunogenic DAMPs signals emitted by dying neurons, chronic neuroinflammation can be induced by activating microglia and astrocytes (Bajwa et al., 2019). On the other hand, in the context of neuroinflammation activation, DAMPs are able to induce phagocytosis of dying cells. For the functioning of the nervous system, an important process in the developing and mature brain is phagocytosis. Neuronal sorting, as a result of which a significant part of neurons dies and must be removed by phagocytes, is an important stage in the development of the brain (Damisah et al., 2020). In the mature brain, under physiological conditions, some unused synapses, periodically dying cells, and emerging cell fragments (debris) must also be regularly removed to maintain homeostasis. Furthermore, in various injuries of the nervous tissue, including ischemic stroke, phagocytosis appears to be especially important for cleaning the area of damage from dead neurons and glial cells and a positive outcome of the inflammatory process (Zhang et al., 2019b). While phagocytosis is mainly associated with microglia (professional phagocytes in the central nervous system (CNS), it may also involve non-professional phagocytes (e.g., astrocytes or oligodendrocytes) (Galloway et al., 2019). It is important to note that in diseases associated with abnormal protein aggregation (e.g., Alzheimer’s disease), phagocytes can engulf protein conglomerates, helping to slow down the progression of the disease (Gabandé-Rodríguez et al., 2020).

Necroptosis has two different outcomes for disease progression. On the one hand, necroptosis promotes cell death and neuroinflammation in several neurodegenerative conditions (Yuan et al., 2019). On the other hand, necroptosis may induce an immune response that prevents tumor progression or creates an immunosuppressive microenvironment (Gong et al., 2019; Mishchenko et al., 2022).

An increase in TNF expression is found mainly in neurons and glia of the region of ischemia after a stroke. Dying cells are also detected in this area. It indicates that stimulation of the Fas/TNFR family induces cell death and then exacerbates cerebral ischemia and reperfusion injuries (Khoury et al., 2020). The findings indicate that RIP3 deletion, MLKL deletion, or necroptotic loss of function is a potential therapeutic strategy for neuroprotection in ischemic stroke (Zhang et al., 2020c).

Microglia, the primary immune cells of the central nervous system, undergo necroptosis in various pathological processes. Activated microglia release pro-inflammatory cytokines and promote nervous cell necroptosis (Jiang et al., 2018). After oxygen-glucose deprivation neurons released proinflammatory IL-18 and TNF and induced M1 polarization of microglia. RIP3-deficient ischemia-exposed neurons secrete anti-inflammatory cytokines IL-4 and IL-10 and induced M2 polarization (Yang et al., 2018a). Thus, RIP3 and MLKL induce microglial polarization toward the M1 phenotype. It was also found that M1 microglia and their receptors damage epithelial cells and destroy the blood–brain barrier (Chen et al., 2019a).

## Necroptosis and astrocytes

Astrocytes are the most abundant glial cells in the human brain. They play an important role in the protection of neurons from various damages and regulation of neurons’ functions (Davila et al., 2013; Khakh and Deneen, 2019). The role and function of astrocytes are thoroughly investigated for many neurodegenerative diseases, as presented in a large number of reviews (Almad and Maragakis, 2018; Li et al., 2019; Chen et al., 2020b; Majo et al., 2020). Astrocytes are critical for normal CNS functions, including maintenance of glutamate and extracellular potassium concentrations and water homeostasis (Sofroniew and Vinters, 2010; Elorza-Vidal et al., 2019; Mahmoud et al., 2019). They play a role in synaptic activity (Philippot et al., 2021; Semyanov and Verkhratsky, 2021), are involved in the synthesis of neurotrophic factors (Mizuta et al., 2001) and neurosteroids (Ubuka and Tsutsui, 2022), episodic memory (Loprinzi, 2019), and adaptation to ischemic injury (Hirayama and Koizumi, 2018; Mitroshina et al., 2020; Song et al., 2021). Astrocytes may limit Alzheimer’s (McAlpine et al., 2021) and Parkinson’s disease (Zhang and Lu, 2021). Thus, astrocyte death has a significant effect on brain damage. It can be the first link in a chain reaction of many cell deaths. For example, necroptotic astrocytes can cause neuronal death (Chen et al., 2021c), contribute to the development of amyotrophic lateral sclerosis (Re et al., 2014), multiple sclerosis (Zelic et al., 2021) and epilepsy (Wu et al., 2021b). Although direct inhibition of necroptosis can prevent neuronal death, the underlying mechanism of neuronal loss during neurodegeneration, caused by necroptotic astrocytes, is unknown. A decrease in the number of astrocytes, which play a nutritional function for neurons, can cause a decrease in nutrient intake and, consequently, the death of neuronal cells.

Neuronal apoptosis significantly increased upon treatment with a conditioned medium taken from astrocyte cultures in which necroptosis was induced. Necroptosis induction was found to alter the production of neurotrophic factors. An increase in the glial cell line-derived neurotrophic factor (GDNF) and brain-derived neurotrophic factor (BDNF) expression in necroptotic astrocytes *in vitro* and an increase in their release of pro-BDNF in extracellular vesicles (EVs-NAS) were revealed. It was demonstrated that the treatment of neurons with such extracellular vesicles has a negative effect on their survival. At the same time, neuronal apoptosis induced by EVs-NAS can be significantly attenuated by blocking pro-BDNF antibodies (Chen et al., 2021c).

It was found that reactive astrocytes first undergo necroptosis after spinal cord injury, while necroptotic astrocytes are less likely to contribute to neuronal survival (Fan et al., 2016). The induction of necroptosis is probably associated with the polarization of M1 microglia/macrophages and the development of chronic inflammation.

A conditioned medium of M1 microglia/macrophages can induce necroptosis of astrocytes. Depletion of microglia *in vivo* significantly reduces necroptosis of astrocytes. This may be due to an increase in the expression of toll-like receptor 4 (TLR4) by microglia polarized toward the M1 phenotype. M1 microglia/macrophages can induce astrocyte necroptosis by activating TLR4 and myeloid differentiation primary response gene 88 (MyD88) signaling. Inhibition of astrocytic necroptosis can restore the neurotrophic function of astrocytes, promoting the survival of neurons surrounding the lesion center. If astrocyte recovery does not occur, neurons undergo apoptosis during secondary injury (Fan et al., 2016).

Astrocytes show significant morphological, molecular, and physiological heterogeneity (Pestana et al., 2020). The morphofunctional heterogeneity of astrocytes and the role of various subtypes in the development of pathologies are currently being actively studied. There are several approaches to the classification of astrocyte subtypes. One of the first approaches was the division into inflammatory (neurotoxic) astrocytes (A1) and neuroprotective astrocytes (A2). In A1 astrocytes, the expression of genes of the classical complement cascade, which has previously been shown to destroy synapses, as well as pro-inflammatory cytokines, etc., is activated. It is assumed that A1 astrocytes can be neurotoxic and have a negative effect on nerve cells in various pathologies. In contrast, many neurotrophic factor genes are activated in A2 astrocytes, suggesting that A2 astrocytes may play a neuroprotective role. Type A1 astrocyte activation is induced by activated microglia during neuroinflammation, trauma, and neurodegenerative processes. In this case, A1 astrocytes lose a significant part of their normal astrocytic functions (the ability to promote the survival and growth of neurons, form synapses, and perform normal phagocytosis); they become neurotoxic, which leads to the death of neurons and oligodendrocytes. A1 astrocytes are formed after CNS damage and in many neurodegenerative diseases (Alzheimer’s disease, amyotrophic lateral sclerosis, and multiple sclerosis). Inhibition of A1 astrocyte formation after acute CNS injury prevents neuronal death. The role of reactive astrocytes in the development of necroptosis in the CNS has only begun to be explored, so there is little data on this topic. For example, in the rat model of transient middle cerebral artery occlusion and the *in vitro* glucose-oxygen deprivation model with reoxygenation, RIP1K levels were significantly increased in reactive A1 astrocytes. RIP1K knockdown or delayed (6-hour therapeutic window), administration of the RIP1K inhibitor Nec-1 suppressed the expression of glial scar markers and reduced infarct volume and neurological deficit (Ni et al., 2018; Zhu et al., 2021b).

The study by Fan et al., 2016 is of particular interest. It shows that astrocytes are the main cell type that undergoes necrosis after spinal cord injury. The critical role of microglia/macrophages M1 in inducing astrocyte necroptosis *in vitro* has been demonstrated. Reducing the population of microglia/macrophage M1 significantly decrease astrocyte necroptosis. In addition, it has been shown that inflammatory responsive genes TLR4 and myeloid differentiation primary response gene 88 (MyD88) are induced in necroptotic astrocytes. Thus, there is an association between microglia/macrophages M1 and necroptosis-type death of astrocytes, which is currently poorly researched. In the future, researchers will have to study in detail the interactions between microglia and reactive astrocytes, as well as investigate in more detail reactive astrocytes of phenotype A2.

Summarizing the available data on the role of necroptotic astrocytes and other types of glial cells in the development of various CNS pathologies, we can say that in recent years a concept postulating that neuroinflammation is one of the key mechanisms responsible for damage to the nervous system and the development of neurodegenerative diseases has been formed. Microglia and astrocytes, which regulate and modify each other’s activity and secretory profiles, and also affect the activity and functioning of neurons, are involved in the development of neuroinflammation. Liddelow et al., 2017 have shown that activated microglia can induce the activation of astrocytes into the A1 phenotype by secreting pro-inflammatory cytokines IL-1α, TNF, and C1q, which leads to neuronal death. Activated microglia alone are insufficient to induce neuronal death, but A1 astrocytes induced by microglia cause neurodegeneration by secreting a neurotoxin and releasing many complement components. In addition, A1 astrocytes inhibit the proliferation and differentiation of oligodendrocyte progenitor cells and lead to the death of mature oligodendrocytes, which plays an important role in the development of demyelinating diseases.

Similar data is available for traumatic brain injury. In mice with traumatic brain injury, progressive chronic damage to the cerebral cortex and hippocampus, as well as an increase in phosphorylated MLKL (pMLKL) levels were observed. In RIPK1- or RIPK3-deficient knockout mice, chronic brain damage significantly declined within 1-3 months, and the volume of injury decreased to 80% (according to magnetic resonance imaging data). At the same time, there was no difference in the severity of symptoms between the knockout and wild-type (WT) mice in the acute period after injury. Neuroprotection in the late period was accompanied by a decrease in the level of astrocyte and microglia activation and an improvement in memory function compared to WT animals. In neuronal RIPK1 deficient mice, there was a decrease in glial scar formation and GFAP expression level. A complete knockout of RIPK1 in all types of nerve cells did not improve motor deficits and memory, although under physiological conditions (without trauma modeling), there were no memory impairments in complete knockouts (Wehn et al., 2021).

However, the binary classification of astrocytes cannot describe the entire diversity of the heterogeneity of different populations of astrocytes (Escartin et al., 2021). For example, transcriptome studies have identified several unique clusters of reactive astrocytes in autoimmune diseases (Wheeler et al., 2020) and Alzheimer’s disease (Habib et al., 2020).

In addition, morphologically different astrocytes in different parts of the brain have been described. Regional heterogeneity of astrocytes has been shown to underlie different responses to treatment for bacterial infections, stroke, and possibly Norrie disease (Gudkov et al., 2023). Probably, further studies will reveal the features of cell death and the role of heterogeneity of astrocytic populations in various CNS diseases.

## Ischemic stroke

Due to its growing incidence, stroke is the leading cause of long-term disability and death (Barthels and Das, 2020). Ischemic stroke accounts for 70-80% of all strokes worldwide, and survivors often have sensorimotor impairment in one or more areas of the body (Barthels and Das, 2020). Ischemia disrupts the blood supply to brain tissue, which subsequently promotes a cascade of pathophysiological reactions leading to various types of cell death, including autophagy, apoptosis, necroptosis, and ferroptosis (Davidson et al., 2020). Necroptosis was shown to cause and exacerbate brain tissue damage after cerebral ischemia and reperfusion injury (She et al., 2020; Zhang et al., 2020c). After ischemic stroke, there is an increase in TNF, whose binding to death receptors TNFR1 contributes to the activation of the molecular cascade controlled by RIP1 (Frank and Vince, 2019).

In ischemic injury, the overall level of damage depends on the damage received not only by neurons but also astrocytes (Nakashima et al., 1995). Despite some evidence that neurons, rather than astrocytes, are primarily exposed to necroptosis in ischemic injury (Han et al., 2019), the role of astrocytes remains underestimated. It is assumed that astrocytes can participate in the restoration of neurons and synaptic connections after cerebral ischemia (Hirayama and Koizumi, 2018). There is evidence that pharmacological inhibition of astrocyte necroptosis by necrostatin-1 (Nec-1) provides neuroprotection against ischemic brain injury. Therefore, RIPK1 can be considered a target for the treatment of cerebral ischemia. Studies by Zhu et al. have demonstrated that the tumor suppressor NDRG2 is involved in the regulation of cerebral ischemic injury and astrocytic necroptosis. NDRG2 knockout led to a significant decrease in GFAP expression levels, and a significant increase in the protein and mRNA expression levels of RIPK1 in astrocytes (Zhu et al., 2020).

## Neurodegenerative diseases

### Alzheimer’s disease

Perhaps the necroptotic death of nerve cells causes the loss of neurons at the early stages of Alzheimer’s disease (AD), when beta-amyloid (Aβ) plaques and neurofibrillary tangles are not yet formed. RIPK1 was found to be activated in brain samples of patients with AD, and there is a correlation between markers of necroptosis and the stage of disease development (Caccamo et al., 2017; Salvadores et al., 2022). One of the proposed mechanisms causing necroptosis in AD is the activation of the TNF/TNFR1 inflammatory cascade. TNF levels are elevated in cerebrospinal fluid and plasma in aging, mild cognitive impairment, and in patients with AD. A high level of TNF is a potential marker of the progression of cognitive impairment to AD. Upon TNF stimulation, accumulated p62 recruits RIPK1, induces its self-oligomerization, and activates the downstream RIPK1/RIPK3/MLKL cascade, leading to neuronal activity necroptosis (Jayaraman et al., 2021; Xu et al., 2021a).

Neuronal necroptosis triggered by TNF is also modulated by autophagy impairment associated with inhibiting the key autophagy signaling regulator UVRAG (Xu et al., 2021a). Activated microglia play an important role in TNF production and subsequent induction of necroptosis (Salvadores et al., 2022). In addition, necroptosis is involved in rod degeneration in AD at an early stage of the disease, even before Aβ plaques are detected in the brain (Zhang et al., 2021b). At the same time, no colocalization of the necroptosis marker pMLKL with astrocytes or microglia was observed in AD (Jayaraman et al., 2021). Inhibition of necroptosis with pharmacological blockers or genome editing agents reduces Aβo-induced neurodegeneration and memory impairment in mice (Caccamo et al., 2017; Salvadores et al., 2022) and zebrafish (Gao et al., 2022).

### Parkinson’s disease

Parkinson’s disease (PD) is a common neurodegenerative disease characterized by the death of midbrain dopamine neurons (de Lau and Breteler, 2006). The etiology of PD is complex and includes the formation of reactive oxygen species and oxidative stress, mitochondrial dysfunction, chronic inflammation, and neuronal death. So far, the pathogenesis of Parkinson’s disease was not well described. Apoptosis was thought to be the primary mechanism of neuronal death in PD, but now there is evidence of an important role of other pathways of regulated cell death, including necroptosis, which probably contribute to neurodegeneration in the disease (Venderova and Park, 2012). The activation of necroptosis in the post-mortem brain tissue of patients with Parkinson’s disease was revealed (Oñate et al., 2020).

It is known that the aggregation of α-synuclein plays a key negative role. Astrocytes in the midbrain play a complex role in PD, initiating both (Chou et al., 2021) and protective (Tsunemi et al., 2020) processes that vary depending on the course of the disease. Interestingly, the accumulation of α-synuclein in astrocytes induces a powerful inflammatory response, and the activated molecular cascade involves RIPK signaling and causes their necroptosis (Oñate et al., 2020). However, it was suggested that, in this case, activation of the RIPK-controlled cascade does not lead to necroptosis, the canonical outcome of this pathway (Chou et al., 2021). Apparently, the action of α-synuclein on astrocytes in PD causes a new, as yet unstudied, type of cell death or a special form of necroptosis.

For example, the study by Chou (Chou et al., 2021) showed that α-synuclein preformed fibrils (PFFs) cause pathogenic activation of human midbrain astrocytes, characterized by inflammatory transcriptional responses, suppression of phagocytic function, and the formation of neurotoxic activity. These effects are mediated by RIPK1 and RIPK3 kinases but are independent of MLKL and, accordingly, the development of necroptosis. Treatment of astrocyte cultures with synuclein led to the activation of genes regulating A1 astrocyte activation. Inhibition of Inhibitory-κB Kinase (IKK) kinase and, consequently, the NF-κB cascade prevented A1 astrocyte activation. Probably, astrocyte activation occurs through RIPK-dependent activation of NF-κB signaling.

The role of increased interaction between RIPK1 and pMLKL and the formation of necrosome complexes in the striatum in axonal neurodegeneration was demonstrated in a toxicological model of PD. However, no such activation of necroptosis was shown in astrocytes [93].

Suppression of necroptosis by Nec-1 reduced 6-hydroxydopamine-induced death of PC12 cells (Wu et al., 2015), protected mice from dopamine cell loss in the modeling of PD using subchronic administration of MPTP (1-methyl-4-phenyl-1,2,3,6-tetrahydropyridine), which causes permanent symptoms of Parkinson’s disease (20 mg/kg MRTR intraperitoneally daily within 5 days), and also increased survival of iPSCs derived from patients with PD (Iannielli et al., 2018). However, the use of a Nec-1s inhibitor did not fully protect against dopaminergic neurodegeneration at subacute concentrations of MPTP (40 mg/kg) (Dionísio et al., 2019).

RIP3 knockout mice (RIP3ko) lacked dopaminergic neurodegeneration in the substantia nigra in a MPTP model of Parkinson’s disease. In addition, these mice showed a decrease in the level of apoptosis, possibly due to the increased levels of the anti-apoptotic protein Bcl-2 in the midbrain but not in its other parts. Moreover, astrocyte cultures obtained from RIP3 knockout animals showed a decrease in mRNA expression of pro-inflammatory cytokines TNF, NLR family pyrin domain-containing protein 3 (NLRP3), and IL-1β after lipopolysaccharide stimulation, which indicates the necroptosis-independent role of RIP3 in the development of inflammation. Interestingly, in RIP3ko mice, increased GFAP production in astrocytes in the midbrain was observed when PD was modeled. It indicates long-term astrogliosis in this area of the brain. However, there was no activation of microglia, which proves a non-inflammatory phenotype of astrocytes. In addition, RIP3ko animals showed normalization of GDNF levels in the midbrain. It is known that the decrease in GDNF production is one of the most important causes of dopaminergic neuron death (D’Anglemont de Tassigny et al., 2015; Chmielarz and Saarma, 2020). Probably, non-inflammatory astrogliosis allowed to normalize GDNF production, thereby enhancing the neurotrophic function of astrocytes and preventing neuronal death of substantia nigra cells (Dionísio et al., 2019).

The study by Yong-Bo Hu et al. (2019) is of particular interest. A specific siRNA, miR-425, which targets RIPK1 transcripts and promotes MLKL phosphorylation and necroptosis, was found to be associated with the death of dopaminergic neurons. Necroptosis activation and miR-425 deficiency in the substantia nigra were observed in mice in a pharmacological model of PD (MPTP) and post-mortem brain tissues. Genetic knockdown of miR-425 in PD modeling exacerbated motor deficits and dopaminergic neurodegeneration through early activation of necroptotic genes. The administration of miR-425 mimetics (AgomiR-425) attenuated the activation of necroptosis and the loss of dopaminergic neurons and also improved the motor functions of experimental animals (Hu et al., 2019).

### Multiple sclerosis

Multiple sclerosis (MS) is a chronic autoimmune disease of the CNS characterized by neuroinflammation, demyelination, and axonal degeneration (Oh et al., 2018; Dobson and Giovannoni, 2019). RIPK1 levels were shown to correlate with multiple sclerosis progression (Zelic et al., 2021). An increase in the expression of RIPK1, RIPK3, and MLKL was detected in post-mortem brain samples of patients (Ofengeim et al., 2015).

In MS, RIPK1 can be activated in microglia, astrocytes, and oligodendrocytes (Dhib-Jalbut and Kalvakolanu, 2015; Ofengeim et al., 2015). RIPK1 activation in microglia and astrocytes *in vitro* induce an inflammatory transcriptional response, which leads to destruction of oligodendrocytes. Interestingly, in MS microglia RIPK1 activity mediates inflammation and cell death, but astrocytes have lower MLKL expression and do not undergo necroptosis. So, differences in expression levels RIPK1, RIPK3, and MLKL and sensitivity level among different cell types may explain the variable susceptibility to death (Zelic et al., 2021).

However, RIPK1 activation mediates a strong inflammatory response in astrocytes, suggesting that RIPK1 can propagate neurotoxic inflammation. This leads to the death of oligodendrocyte precursors and triggers demyelination processes. Therapeutic administration of a RIPK1 inhibitor to mice attenuates the disease phenotype in a dose-dependent manner (Zelic et al., 2021).

### Amyotrophic lateral sclerosis

Amyotrophic lateral sclerosis (ALS) is a fatal neurodegenerative disease characterized by the loss of both cortical and spinal motor neurons. Patients diagnosed with ALS initially experience muscle weakness or convulsions, eventually leading to paralysis and death, usually within 3–5 years (Wijesekera and Nigel Leigh, 2009). ALS is often associated with mutations in superoxide dismutase 1 (SOD1). Glial cells, primarily astrocytes, determine the rate of disease progression in SOD1-mutated mice, which are an experimental model of ALS (Yamanaka et al., 2008). Astrocytes obtained from SOD1 mutant mice were selectively toxic to mouse and human motor neurons (Nagai et al., 2007; di Giorgio et al., 2008), as were astrocytes obtained from patients with various forms of ALS (Haidet-Phillips et al., 2011). At the same time, it is noted that the death of motor neurons caused by astrocytes obtained from patients with ALS occurs through necroptosis (Re et al., 2014). Thus, it is likely that astrocyte necroptosis initiates neuronal loss in ALS.

Most authors agree that necroptosis plays a key role in motor neuron degeneration in amyotrophic lateral sclerosis (Haidet-Phillips et al., 2011). A number of studies demonstrated an increase in the expression of key signaling markers of necroptosis (RIPK1, RIPK3, and MLKL) both in mouse models and post-mortem spinal cord tissues of patients with sporadic ALS (Ito et al., 2016). However, some studies do not support these findings. For example, in a study by Wang (Wang et al., 2020), in mice in which the MLKL gene was deleted to eliminate this critical effector of necroptotic cell death, the manifestation, progression, and outcome of the disease did not change. Thus, the role of necroptosis in ALS is controversial and needs further research.

### Epilepsy

Epilepsy is a multi-etiologic disease that has many forms. It is a chronic relapsing brain disease with a high incidence, up to 1-2% of the population. Medial temporal lobe epilepsy is the most common and severe form of focal epilepsy in adults. This form of epilepsy is characterized by hippocampal sclerosis. In recent years, there has been increasing evidence that dysfunctional astrocytes play a crucial role in the development of medial temporal lobe epilepsy. In hippocampal sclerosis, there is often a massive loss of neurons. Astrogliosis is also observed, especially in the hilus of the dentate gyrus and in the CA3 and CA1 regions of the hippocampus, which indicates the role of dysfunctional microglia and astrocytes in disease initiation.

It was shown that after pilocarpine- or kainate-induced status epilepticus, mice have a significant decrease in the density of astrocytes in the CA1 stratum radiatum (SR) of the hippocampus (Kim et al., 2010). In addition, an increase in the expression of RIPK3 and MLKL in astrocytes was demonstrated. Four hours after SE induction, necrosome complexes and translocation of phosphorylated MLKL to the nucleus and plasma membrane were detected. At the same time, there was no activation of caspases and genes associated with apoptosis. The production of pro-inflammatory cytokines, including TNF, probably plays a critical role in the induction of necroptosis in status epilepticus. Subsequently, the number of astrocytes was restored to the initial values, which was accompanied by increased proliferation (Wu et al., 2021b).

In neurons, an increase in the expression of RIP3 and p-MLKL was also noted in the acute period after status epilepticus (Scharfman and Buckmaster, 2014; Cai et al., 2017). The maximum expression was observed after 72 hours, after 7 days, it decreased. In the amygdala and piriform cortex, cells that die through necroptosis and express MLKL on the plasma membrane were found (Cai et al., 2017).

Injection of necrostatin-1 into the cerebral ventricles reduced damage to hippocampal tissue in mice in a kainate model of epilepsy. In addition, necrostatin-1 significantly suppressed necroptosis-associated proteins (MLKL, RIP1, and RIP3) and apoptosis-associated proteins (cleaved caspase-3, apoptosis regulator BAX) and increased the expression of the anti-apoptotic Bcl-2 protein (Lin et al., 2020a).

### Huntington’s disease

Huntington’s disease (HD) is an autosomal dominant genetic disease and a progressive disorder of motor, cognitive and mental functions. After diagnosis, the median life expectancy is 15 to 18 years (Caron et al., 1993). The disease is caused by an abnormal expansion of CAG trinucleotide repeats in the huntingtin (HTT) protein gene on chromosome 4, which prevents proper protein folding (Bates et al., 2015). The function of normal HTT remains unclear. However, it is assumed that it can be involved in selective autophagy (Ochaba et al., 2014) and mitophagy (Franco-Iborra et al., 2021). In addition, HTT is able to interact with the cytoskeleton (Taran et al., 2020). However, it has long been known that mutant huntingtin (mHTT) causes the formation of insoluble aggregates, acquires the ability to interact with new proteins, affects the regulation of gene transcription, and stimulates the loss of mitochondrial function (Cisbani and Cicchetti, 2012). It results in the loss of neurons, which is especially noticeable in the structures of the striatum, caudate nucleus, and putamen (MacDonald et al., 1993). To date, the main way to maintain the quality of life of patients with HD is the reversal of manifestations using pharmacological therapy, antipsychotics, and psychotropic drugs (Caron et al., 1993).

It is assumed that inhibition of necroptosis in HD can prevent cell death and partially reverse the symptoms of the disease. For example, transplantation of stem cells into the degenerated striatum can protect neurons and improve locomotor activity in a rat model of Huntington’s disease. This fact has been associated with the inhibition of necroptosis (Bayat et al., 2021). Moreover, the use of the necroptosis inhibitor Nec-1 can extend lifespan and delay the onset of disease in HD mice (Zhu et al., 2011). However, an increase in RIPK1 expression induces mitophagy and alleviates neurodegeneration (Khalil et al., 2015). Nevertheless, there is very little data on the role of necroptosis in the development of Huntington’s disease, so more in-depth studies are required.

### Spinocerebellar ataxias

Spinocerebellar ataxias (SCAs) are a group of autosomal hereditary diseases, including more than 40 types. The prevalence of this group of diseases is about 1:10 000 (Ruano et al., 2014). Some types of SCAs affect the cerebellum and spinal cord, but in most cases, SCAs are characterized by damage to other parts of the brain, such as the brainstem and basal ganglia. Genetically, SCAs are divided into two groups: SCAs caused by a triplet repeat expansion are more common, and SCAs caused by non-repeat mutations are less common (Klockgether et al., 2019). The clinical manifestations of SCAs mainly include impaired sense of balance and coordination, dysarthria, vision problems, and cognitive impairment (Sullivan et al., 2019). There are few studies on the role of necroptosis in this group of diseases. It has been shown that transgenic mice with the Atxn2-CAG100-KIN model of ataxia demonstrate an increase in the expression of RIPK1 (Canet-Pons et al., 2021) and RIPK3 (Murru et al., 2019), which contributes to the activation necroptosis in SCA types 2 and 28, respectively. The activation of ERK1/2-RIP3 and ERK-MSK1 cascades, which promote neurodegeneration in spinocerebellar ataxia type 1 (SCA1), has a similar effect (Park et al., 2013). Thus, it can be assumed that inhibition of necroptosis can help correct the manifestations of SCAs. For example, Saeidikhoo et al. have already shown that implantation of Sertoli cells has a neuroprotective effect and improves motor function in animals by counteracting necroptosis in cerebellar ataxia (Saeidikhoo et al., 2020). However, there are almost no such pilot studies, and in the future, researchers will have to study this issue in detail.

### Sphingolipidoses

Sphingolipids are amphiphilic molecules that are poorly soluble in water and aggregate in aqueous solutions (Sandhoff, 2013). Sphingolipidoses are a class of hereditary lysosomal storage diseases in which sphingolipids accumulate in one or more organs as a result of a primary deficiency of enzymes or activator proteins involved in their degradation pathway. Another name for such diseases is lysosomal storage disorders (LSDs). Thus, these are diseases characterized by the accumulation of macromolecules in the late endocytic system. They are caused by hereditary defects in genes encoding mainly lysosomal enzymes or transmembrane lysosomal proteins (Yañez et al., 2020). Depending on the defect, various cells can be the target of lysosomal accumulation, for example macrophages are a common target, but neuronal cells, endothelial cells, cardiomyocytes, etc. can also be affected (Vanier et al., 2016). Normally, sphingolipids are catabolized in lysosomes by specialized proteins. Mutations in certain genes form stable micelles with a molecular weight of more than 1000 kDa (Hollak, 2022).

There is no therapy for most sphingolipidoses, especially those that are typical for the nervous tissue, however, enzyme replacement therapy is currently used for some diseases (Sandhoff, 2013). The most common sphingolipidoses resulting in brain damage are Niemann-Pick disease, Krabbe disease, and Gaucher disease. In a mouse model of Krabbe disease, an increase in RIPK1 expression in microglial cells and activation of cascades controlled by it, which lead to neuroinflammation, were found. It is interesting to note that RIPK1 knockout in twitcher mice (a line for modeling Krabbe disease) did not slow down the disease progression, and the symptoms did not differ from normal twitcher mice, which, apparently, may be due to the feature of the line where genes downstream in the RIPK1 cascade are already defective, so its contribution is unnoticed (Cachón-González et al., 2021). It is also known that RIPK1 and RIPK3 expression levels are increased in the brains of mice with Gaucher disease and Niemann-Pick disease (Vitner et al., 2014; Cougnoux et al., 2016). An increase in RIPK1 was observed in the brain of a person who died of HD (Vitner et al., 2014), and increased expression of RIPK3 and MLKL was found in macrophages derived from stem cells from patients with Gaucher disease, indicating that necroptosis plays a role in triggering inflammation-related cell death in Gaucher disease (Messelodi et al., 2021).

Although necroptotic cell death has been shown to develop in these diseases, the mechanisms of its activation are still undetermined. One of the possible pathways is the interaction of tyrosine kinase c-Abl with RIPK3. When c-Abl is inhibited, a decrease in RIPK3 signaling has been observed in models of Gaucher disease. It has also been found that c-Abl mediates RIPK3 phosphorylation in the activation site (Yañez et al., 2021). To date, in clinical practice, there are no registered drugs for the treatment of sphingolipidoses, the main action of which is the inactivation of necroptosis. However, some studies can initiate the creation of such drugs. For example, it has been shown that the effect on necroptosis can be a potential therapeutic target, at least in Niemann-Pick disease (Cougnoux et al., 2018).

### Frontotemporal dementia

Frontotemporal dementia (FTD) is the second most common cause of early dementia after AD, manifesting itself before the age of 65 (Olney et al., 2017). Frontotemporal dementia has both sporadic forms and genetically determined causes since about 40% of cases have a family origin (Shpilyukova et al., 2020). FTD is characterized by the deposition of abnormal DNA-binding protein 43 (TDP-43) or tau protein aggregates in the frontal and temporal lobes of the brain, resulting in neuronal degeneration, behavioral abnormalities, or language dysfunction with progressive degeneration of the frontal and temporal lobes. Dobson-Stone et al. demonstrated that an overactive CYLD M719V mutant gene could contribute to the pathogenesis and development of frontotemporal dementia. It might at least in part lead to neurodegeneration via inappropriate necroptosis of neurons (Dobson-Stone et al., 2020a,b). The pathogenesis of FTD is associated with changes in the TAR DNA-binding protein 43 (Carlos and Josephs, 2022). It has been shown that in this type of dementia, TDP-43 undergoes additional ubiquitination (Arai et al., 2006). It has also been found that a 1.6- and 2.5-fold increase in the expression of RIPK1 and MLKL, respectively, occurs through the TDP-43 activation, which suggests the activation of necroptosis in FTD (Wang et al., 2018). In parallel, there is a decrease in the expression of the endogenous RIPK1 inhibitor TANK-binding kinase 1 (TBK1), which leads to neuroinflammation, additional TDP-43 aggregation, axonal degeneration, loss of neurons and behavioral deficits, and the further manifestation of signs of ALS and FTD (Freischmidt et al., 2017). Thus, necroptosis plays an important role in the pathogenesis of FDT.

### Depression

Major depressive disorder (MDD) according to the DMS-5 classification or depressive episode and recurrent depressive disorder according to ICD-10, 11, is a widespread mental illness, often associated with stress, which affects more than 300 million people worldwide. MDD entails severe consequences, secondary morbidity, and even death (Walker et al., 2015). Depression is a polyetiological disease with an incompletely investigated etiology. Over the past 2-3 years, the first evidence of the correlation between the pathogenesis of mental disorders, including depression, and the development of necroptosis has appeared. For example, an increase in the expression of RIPK1, RIPK3, MLKL, and p-MLKL was observed when modeling chronic unpredictable mild stress in animals and developing depressive-like behavior. The administration of Nec-1 reduced behavioral changes and reduced the development of neuroinflammation (He et al., 2021).

The study by Sun et al., 2022 showed that the deletion of nerve injury-induced protein (Ninj2) in oligodendrocytes in mice inhibits their development and myelination and leads to an impaired neuronal morphology and activity, which is of great interest. Ninj2 competitively inhibits the TNF/TNFR1 signaling pathway by binding directly to TNFR1 in oligodendrocytes. Ninj2 knockout activates TNF-induced necroptosis and increases the production of C-C motif chemokine ligand 2 (Ccl2), which may mediate signal transduction from oligodendrocytes to neurons. The use of Nec-1s to inhibit necroptosis restored the development of oligodendrocytes, improved neuronal excitability, and alleviated depressive-like behavior in animals.

Sepsis-associated encephalopathy (SAE) is a complication of sepsis that leads to mental health problems and depression. Chronic depressive-like behavior, as well as a significant increase in neuronal apoptosis and necroptosis, were detected in mice with a sepsis model by cecal ligation and puncture (CLP). Inhibition of myeloid differentiation factor 2 (MD2), a mediator of both apoptosis and necroptosis, reduced neuronal death and improved depressive behavior in animals (Fan et al., 2022). Summing up, necroptosis and RIPK1 may also be a target for the correction of depressive disorders.

**FIGURE 2:**
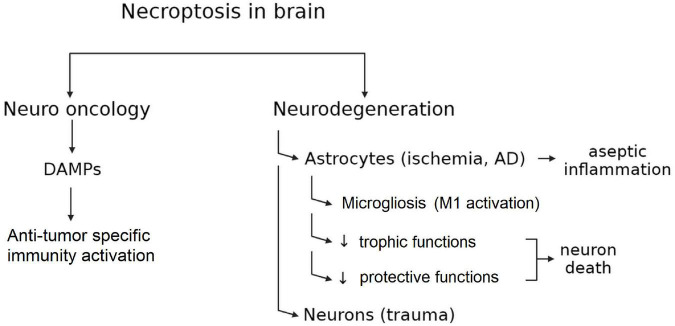
Double-Edged sword of necroptosis in CNS.

Thus, the role of necroptosis for the organism as a whole and for the brain in particular is dual. On the one hand, necroptotic death causes the loss of neurons in ischemic and neurodegenerative diseases. On the other hand, necroptosis may induce an immune response that is extremely important for preventing tumors formation (Figure 2).

Table 1 summarizes the data on the activation of necroptosis in neurons and astrocytes in various CNS pathologies.

**TABLE 1:** Dynamics of the components of the necroptosis pathways involved in various CNS pathologies.

Pathology	Neurons		Astrocytes		Microglia		Inflammation	References
	**Increase**	**Decrease**	**Increase**	**Decrease**	**Increase**	**Decrease**	**Increase**	
Ischemic stroke	RIPK1, RIPK3, pMLKL	MLKL	RIPK1, VEGFR-3, RIPK3, MLKL, pMLKL		RIPK3		TNF, IL-1β, iNOS, IL-6, TRAIL, FasL, ROS, p47, gp91, phox	Qu et al., 2016; Hirayama and Koizumi, 2018; Koizumi et al., 2018; Chen et al., 2019a; Zhu et al., 2020, 2021b; Lule et al., 2021; Wang et al., 2021
Trauma	RIP1, GRP78, CHOP, RIPK3, MLKL, PARP	p-AMPKα, SOD1, Nrf-2	p-NF-κB, NLRP3, ASC, IL-1β, IL-6		RIPK3, p-MLKL		TNF, IL-1β, IL-6, RIP1, p-p65, NF-κB	Lau and Yu, 2001; Liu et al., 2018; Bao et al., 2019; Chen et al., 2020a; Zhao et al., 2020; Hu et al., 2021
Alzheimer’s disease	pRIPK3, pMLKL, pRIPK1, TNFR1, TNFR2, RIPK3, MLKL, MEG3	CASP3, cIAP1, cFLAR	RIPK1, GFAP		Iba1, YM1	Arg1	ROS, TNF, IL-1β, IL-6, iNOS	Koper et al., 2020; Jayaraman et al., 2021; Park et al., 2021
Parkinson’s disease	p-RIPK3, p-MLKL, RIPK1, RIPK3, MLKL, ROS	GDNF, ATP	pMLKL		pMLKL		TNF, IL-1β, IL-6, NLRP3	Dionísio et al., 2019; Alegre-Cortés et al., 2020; Lin et al., 2020b; Oñate et al., 2020
Multiple sclerosis	TNFR1, RIPK1, pMLKL, pRIPK3, FADD	Ñaspase3	RIPK1, CXCL1, CXCL2, CCL2, RGS1		RIPK1, RIPK3, MLKL, CXCL1, CXCL2	CX3CR1, Siglech	TNF, iNOS, IL-6, CXCL1, CXCL2, CCL2, CCL3, CCL4, IFNγ, Mir155hg, Bhlhe40	Yuan et al., 2019; Picon et al., 2021; Zelic et al., 2021
Epilepsy	MLKL, p-MLKL, RIPK3	Ñaspase3	MLKL, LC3B, Ñaspase8, TRAP1, HSP90α, RIPK3	Kir4.1	pMLKL, Iba1		HMGB1, TNF, IL-6	Jia et al., 2019; Wu et al., 2021b; Huang et al., 2022

## Viral, bacterial and parasitic diseases

Necroptosis can be caused not only by internal diseases and injuries but also by parasites and viruses. For example, infection with the parasitic nematode Angiostrongylus cantonensis is the leading cause of eosinophilic meningitis in the Pacific and Caribbean (Guerino et al., 2017). Extensive brain damage is observed during infection. The pathogenesis is still poorly understood, but apoptosis and necroptosis were found to occur in microglia, astrocytes, and neurons after infection with A. cantonensis. Thus, treatment with inhibitors of apoptosis and necroptosis in combination with antiparasitic drugs may be a promising treatment strategy (Mengying et al., 2017).

Necroptosis may play a key role in pathogenesis and has an impact on viral replication in viral diseases. Previously, necroptosis was mainly studied in the context of response to microbial or viral action. It was found that some bacteria and viruses are able to block necroptosis, acting, for example, on RIPK1 (Li et al., 2013), on the DNA-dependent activator of interferon regulatory factors (DAI, also known as ZBP1 or DLM-1)-RIPK3 complex (Upton et al., 2012), RIPK1/RIPK3 (Upton et al., 2008) or TIR domain-containing adapter-inducing interferon-β (TRIF)/RIPK3 (Kaiser et al., 2013). Herpesviruses encode inhibitors that block both caspase-dependent apoptosis and receptor-interacting protein (RIP) kinase-mediated necroptosis (Yu et al., 2016). Cowpox virus actively inhibits necroptosis (Liu et al., 2021c). Influenza A virus can block the course of apoptosis and necroptosis in the cell, turning it into a “factory” for making copies of itself (Balachandran and Rall, 2020), and orthopoxviruses have developed a mechanism that helps block necroptosis in the host cell (Liu et al., 2021c). The fact that viruses during evolution devolved the ways to avoid cell necroptosis indicates the importance of this pathway in combating microbial infection (Bertheloot et al., 2021).

Hepatitis C virus-induced apoptosis blockade by blocking caspase 8 in human embryonic kidney 293T cells (Prikhod’ko et al., 2004), however, it induces necroptosis as well by activating RIPK3 (Jung et al., 2020). Influenza A virus (Zhang et al., 2020b), Coxsackievirus A6 (Zhang et al., 2020a), respiratory syncytial virus (Simpson et al., 2020), and the bacterial Salmonella effector SpvB (Dong et al., 2022) can also induce necroptosis by activating RIPK3 and MLKL that has been shown at primary wild-type murine embryo fibroblasts (Zhang et al., 2020b), human rhabdomyosarcoma cells, human embryonic kidney cells 293T cells (Zhang et al., 2020a), human and murine airway epithelial cell (Simpson et al., 2020), human colorectal adenocarcinoma cells CaCo-2 and Hela (Dong et al., 2022).

Astrocytes may be the main target for various neurotropic viruses such as Zika virus (van den Pol et al., 2017; Wen et al., 2021) or they may act in as a repository for the human immunodeficiency viruses HIV-1 (Khan et al., 2022). Zika virus (ZIKV) is a flavivirus spread by blood-sucking insects, mainly mosquitoes. Zika virus can cause neurological disorders in humans. It is transmitted through the placental barrier and can cause severe fetal developmental disorders: neurodevelopmental disorders and microcephaly. Necroptosis of astrocytes was found to be triggered by viral load, while apoptosis or pyroptosis of astrocytes is not observed in the Zika virus (Wen et al., 2021). When neurons are infected with the Zika virus, RIPK signaling is activated, which limits virus replication (Daniels et al., 2019), however, it does not always lead to the activation of necroptosis and cell death (Daniels et al., 2017). The choice of the type of cell death in infected astrocytes is of particular interest. Necroptosis is usually avoided as it causes extensive inflammation, which can lead to dire consequences (Orozco and Oberst, 2017). The virus probably blocks other safer ways of programmed death, so the only process left for the cell is necroptosis (Wen et al., 2021). Interestingly, inhibition of RIPK3 by GSK’872 reduces necroptotic astrocyte death in Zika virus, whereas necrostatin-1 (an inhibitor of RIPK1) has no protective effect, suggesting that ZIKV-induced necroptosis in astrocytes is independent of RIPK1. Perhaps, in this case, the induction of necroptosis is mediated by the activation of the nucleotide cytoplasmic sensor of the interferon-inducible protein Z-DNA binding protein 1, which can activate RIPK3 (Wen et al., 2021). ZBP1 also plays a role in HSV-1 restriction and further initiation of cell death by apoptosis or necroptosis, reducing the release of viral particles (Jeffries et al., 2022). New mediated pathways for the influence of viruses on the activation of necroptosis can also be identified. For example, it is known that viral infection is often accompanied by the secretion of TNF (Gyurkovska and Ivanovska, 2016). In turn, TNF can induce necroptosis and apoptosis in neurons and astrocytes, respectively (Zhou et al., 2022).

**FIGURE 3:**
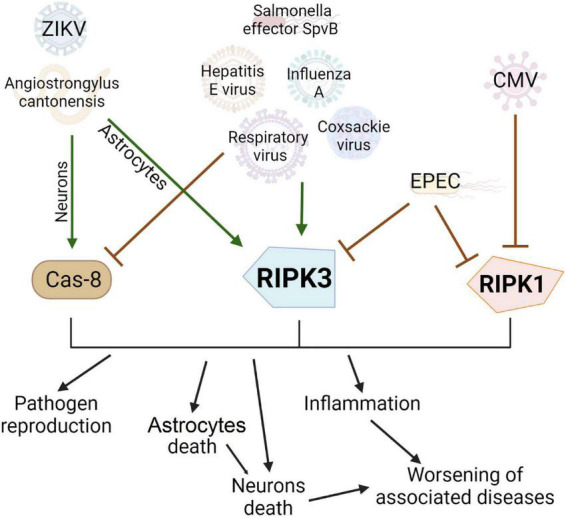
Effect of viruses, bacterial and other pathogens on the development of necroptosis and apoptosis. Pathogens can be conventionally divided into three groups: activating apoptosis, activating necroptosis, or blocking cell death. Many pathogens, such as the hepatitis E virus, have developed evolutionary adaptations to prevent apoptosis by blocking Caspase 8. In this case, the infected cell will live longer and more copies of the virus will be produced. However, this contributes to the activation of another type of cell death, necroptosis, via RIPK3 activation. Enteropathogenic E. coli can inhibit the main proteins of necroptosis and prevent this type of cell death. Interestingly, the Zika virus causes necroptosis in the cell, which limits the replication of the virus and has no apparent evolutionary advantage but causes extensive inflammation in the brain.

Viruses, bacteria, and parasites have a multidirectional effect on the course of necroptosis in different cells of the body, influencing both activation of death and its inhibition which depends on the type of infection and associated conditions (Figure 3).

## RIP kinase inhibitors and their potential use in the treatment of CNS diseases

To date, seven members of the RIPK family have been identified. Kinases of this group are important mediators of cell survival and cell death programming (Cuny and Degterev, 2021) and are promising molecular targets for therapeutic effects. Apparently, RIPK1 and RIPK3 play a key role in the development of necroptosis. Therefore, in this section of the review, we will focus on inhibitors of these kinases. More than 40 RIPK1 inhibitors and more than 10 RIPK3 inhibitors have already been developed (Martens et al., 2020; Zhuang and Chen, 2020).

### RIPK1 Inhibitors

RIPK1 appears to be an important target in the pharmaceutical industry, not only because of its key role in TNF signaling responses. The kinase structure of RIPK1 is well suited for the development of specific pharmacological small molecule inhibitors. No pharmacological inhibitor of RIPK1-mediated cell death is currently used in clinical practice (Moerke et al., 2019), but there are both ongoing and completed clinical trials that will be discussed further. To date, many low-molecular compounds that have an ability to inhibit RIPK1 have been identified. Let us consider the inhibitors most used in the scientific literature, and compounds undergoing clinical trials.

(Nec-1), 5-(indol-3-ylmethyl)-3-methyl-2-thio-hydantoin, was the first RIPK-1 inhibitor developed in 2005 (Degterev et al., 2005). It is currently the most widely used RIPK inhibitor in the scientific literature and is considered the gold standard for published articles. There were studies conducted on the potential therapeutic effect of Nec-1 in many diseases affecting all organ systems. Briefly, the neuroprotective effect of necrostatin in ischemic injuries (Liu et al., 2019a; Mitroshina et al., 2022), hydrocephalus (Liu et al., 2021a), mitochondrial dysfunction (Apaijai et al., 2021), intracranial hypertension (Qian et al., 2022) was revealed. Necrostatin reduces postoperative cognitive dysfunction (Yin et al., 2022), neonatal neurotoxicity (Xu et al., 2021b), and cardiotoxicity (Erdogmus Ozgen et al., 2022). Necrostatin reduces osteoblast death after glucocorticoid treatment (Feng et al., 2018), reduces fibrosis of the fat graft, a severe complication after transplantation (Chen et al., 2021b), stimulates hair growth (Zheng et al., 2020) and interstitial pulmonary fibrosis (Mou and Mou, 2020), reduces jejunal injury and improves digestive and barrier function in intestinal injury (Liu et al., 2021b). There is conflicting data on the role of necrostatin in neurodegenerative diseases. According to the results of some studies, the use of Nec-1 does not affect the death of rotenone-exposed neurons in a model of Parkinson’s disease and also does not fully protect against neurodegeneration when modeling PD using MPTP (Dionísio et al., 2019; Alegre-Cortés et al., 2020). On the other hand, necrostatin protects dopaminergic neurons differentiated from fibroblasts of PD patients (Iannielli et al., 2018), suppresses cell death in models of Alzheimer’s disease (Yang et al., 2019; Gao et al., 2022) and Parkinson’s disease (Wu et al., 2015; Iannielli et al., 2018; Lin et al., 2020b).

Despite the fact that there are many studies using Nec-1 as an inhibitor of RIPK1, there is some evidence that it is not selective and is involved in the inhibition of RIPK3 and MLKL (Lin et al., 2020b; Xu et al., 2021a).

Phenytoin is a compound structurally similar to necrostatin. It was registered as an antiepileptic drug and has the second name Phenhydan. Phenhydan was investigated as a potential therapeutic agent for the treatment of early epileptic encephalopathies (Sanofi, 2021a). It was shown to be a potent inhibitor of necroptosis and apoptosis. For example, Phenytoin was shown to attenuate RIPK1 activity *in vitro* and block the upstream steps of necrosome formation in cells undergoing necroptosis (von Mässenhausen et al., 2018). Phenhydan blocked the activation of necrosome formation/activation as well as death receptor-induced NF-κB signaling by affecting cell membrane function such as lipid raft formation (Moerke et al., 2019). Phenhydan appears to act as a death receptor signaling modulator by affecting the properties of the plasma membrane’s lipid bilayer, thereby modifying the behavior of several membrane-associated proteins, including TNFR1. In this regard, studies on the expanded use of this drug as a potential treatment for ischemic brain injuries and a number of neurodegenerative diseases are of great interest (Moerke et al., 2019).

A number of inhibitors are currently undergoing clinical toxicity testing. Their therapeutic effect in various diseases is being studied. The RIPK-1 inhibitor DNL788 or SAR443820 is being investigated as a treatment for multiple sclerosis and its pharmacokinetic parameters in healthy subjects (Sanofi, 2021b). Efficacy of the SAR443122 inhibitor in cutaneous lupus erythematosus (Sanofi, 2022b) and immunomodulatory properties in patients with severe COVID-19 (Sanofi, 2022a) are being evaluated. The effect of GFH312 and GSK2982772 inhibitors on healthy individuals is being studied. They are considered a treatment for inflammatory conditions (GlaxoSmithKline, 2018, 2019a,b; GenFleet Therapeutics, 2022). The efficiency of the GSK2982772 inhibitor in patients with psoriasis (GlaxoSmithKline, 2020b,2021a), rheumatoid arthritis (GlaxoSmithKline, 2021b), and ulcerative colitis (GlaxoSmithKline, 2020a) is of particular interest to researchers. The RIPK1 inhibitor GSK3145095 was tested for its ability to modulate the immune response by sensitizing tumors to checkpoint blockade (Harris et al., 2019). The SAR inhibitor 443060 (DNL 747) was tested in patients with Alzheimer’s disease and amyotrophic lateral sclerosis (Sanofi, 2022c). In addition, there is an active search and identification of new highly selective inhibitors of RIPK1. For example, several new inhibitors have been described in the first quarter of 2022 (Delehouzé et al., 2022; Li et al., 2022b; Niu et al., 2022).

### RIPK3 inhibitors

RIPK3 may be a more promising target for therapy. The inhibition of necroptosis pathway at the level of RIPK3 rather than RIPK1 is expected to be more specific and provide a greater therapeutic effect. There are currently studies on the role of RIPK3 in the formation of acute respiratory distress syndrome in neonates (Daping Hospital and the Research Institute of Surgery of the Third Military Medical University, 2015). Earlier the researchers assessed the level of RIPK3 as a predictor of mortality in patients with sepsis (Universitas Sriwijaya, 2019). However, compared to RIPK1, a much smaller number of selective inhibitors was identified for RIPK3. This may be due to the fact that inhibition of RIPK3 kinase activity can induce apoptosis (Kaiser et al., 2013).

GSK’872 is the most used RIPK3 inhibitor. A search on pubmed reveals 46 articles published in the last 5 years describing the results of RIPK3 inhibition using GSK’872. It was shown to have a neuroprotective effect in hydrocephalus (Liu et al., 2021a) and to affect reducing collagen expression in fibroblasts *in vitro* when modeling fat graft fibrosis (Chen et al., 2021b). GSK’872 decreases neurobehavioral brain defects and edema in intracranial hemorrhage (Chen et al., 2018), protects from emphysema, suppresses the lung inflammation (Chen et al., 2021a), prevents from heat stress-induced intestinal cell death (Li et al., 2020), alleviates liver damage caused by non-alcoholic fatty disease (Zhang et al., 2021a), and reduces astrocyte death caused by Zika virus (Wen et al., 2021). The triple combination of inhibitors GSK’872, GW806742X (MLKL inhibitor), and IDN-6556 (pancaspase inhibitor) demonstrated enhanced inhibition of the necrosis in gout (Zhong et al., 2021).

Dabrafenib is considered a promising therapeutic agent for the treatment of melanoma (Robert et al., 2015; Dummer et al., 2020), lung cancer (Shimada et al., 2021; Novartis, 2022), thyroid carcinoma (Wang et al., 2019; Brandenburg et al., 2021; Saint Petersburg State University, 2021) and solid tumors (Geoerger et al., 2018; Js InnoPharm, 2022). In addition, when using this RIPK3 inhibitor, a neuroprotective effect was shown in mice subjected to ischemic brain injury by photothrombosis (Cruz et al., 2018), it also has potential for the treatment of renal ischemia (Liu et al., 2020). It promotes recovery after brain injury (Sugaya et al., 2019) and is considered a drug for the treatment of Parkinson’s disease (Uenaka et al., 2018). *In silico* drug screening by using genome-wide association study (GWAS) data repurposed dabrafenib, an anti-melanoma drug, for Parkinson’s disease (P3.8-031) (Uenaka et al., 2018).

The use of GSK’840 and GSK’872, selective RIPK3 inhibitors (Ali and Mocarski, 2018) may have a therapeutic effect in the treatment of melanoma (Geserick et al., 2015) and intestinal tumors (Chen et al., 2018). They protect against emphysema (Chen et al., 2021a) and attenuates brain damage from hemorrhage (Chen et al., 2018). TAK-632 was described as a Pan-Raf kinase inhibitor (Okaniwa et al., 2013). Later it was identified that it also targets RIPK3 and RIPK1 (Chen et al., 2019b). An improved TAK-632 with high selectivity for RIPK1 has now been developed and is a promising candidate for ulcerative colitis therapy (Zhu et al., 2021a). Zharp-99, a potent inhibitor of RIPK3 kinase, has the potential for further development of new approaches for treating necroptosis-associated inflammatory disorders (Xia et al., 2020). In general, much attention is now being paid to the synthesis and testing of new RIPK3 inhibitors (Zhang et al., 2019a; Xia et al., 2020; Wu et al., 2021a).

It should be noted that despite the great interest of researchers in the protective effect of necroptosis inhibitors in various pathologies, there are very few works devoted to studying necroptosis as a therapeutic target in neurodegenerative processes. Moreover, the inhibition of necroptosis in specific cell types, primarily astrocytes, seems to be very promising.

## Conclusion

In recent years, our understanding of the existing types of cell death has expanded significantly. Necroptosis is a recently described type of cell death that has similarities with necrosis but is mediated by fundamentally different molecular pathways. In the present review, we summarized the latest experimental data on the role of necroptosis in the pathogenesis of cerebral ischemia and neurodegenerative diseases as an important pathogenetic component of neuronal and especially astrocyte death. Over the past decades, all the attention of neurobiologists and clinicians has been focused on neurons in the study of molecular and cellular mechanisms of development of various diseases of the central nervous system. However, this approach to date does not allow correcting neurodegenerative changes, and hundreds of research groups have failed in the search for new therapeutic targets. Up to date, astrocytes are the hope of researchers around the world. The data we collected indicates that the death of astrocytes through the necroptotic pathway plays an important role in the development of neurodegenerative processes and is involved in the activation of neuronal death. Attempts to use inhibitors of necroptosis, primarily RIPK1 and RIPK3 kinases, to correct ischemic damage and neurodegeneration are of considerable interest. Many works demonstrate significant improvements in the inhibition of necroptosis when modeling pathologies both *in vitro* and *in vivo*.

However, we must remember that the role of necroptosis in the CNS is multifaceted. It plays an important physiological role in the control of neuroinfections, immunological control of carcinogenesis, and other aspects of CNS functioning. Therefore, the use of inhibitors of necroptosis should be carefully and comprehensively investigated, including with respect to long-term effects.

## Author contributions

EM and MS wrote the manuscript. EM and MV provided the idea. MV revised the manuscript. All authors read and approved the final manuscript.
